# Supplementary material for: Improving atlas-scale single-cell annotation models with hierarchical cross-entropy loss
Source: Nat Comput Sci. 2026 Jan 30;6(3):243–9. doi: 10.1038/s43588-025-00945-z (PMC13021517; doi:10.1038/s43588-025-00945-z)
Supplement: Supplementary file 1 — Supplementary Figs. 1–10 and Tables 1–3. [file 43588_2025_945_MOESM1_ESM.pdf]

nature computational science

Supplementary information https://doi.org/10.1038/s43588-025-00945-z

Improving atlas-scale single-cell annotation models with hierarchical cross-entropy loss

In the format provided by the authors and unedited

Supplementary Figures

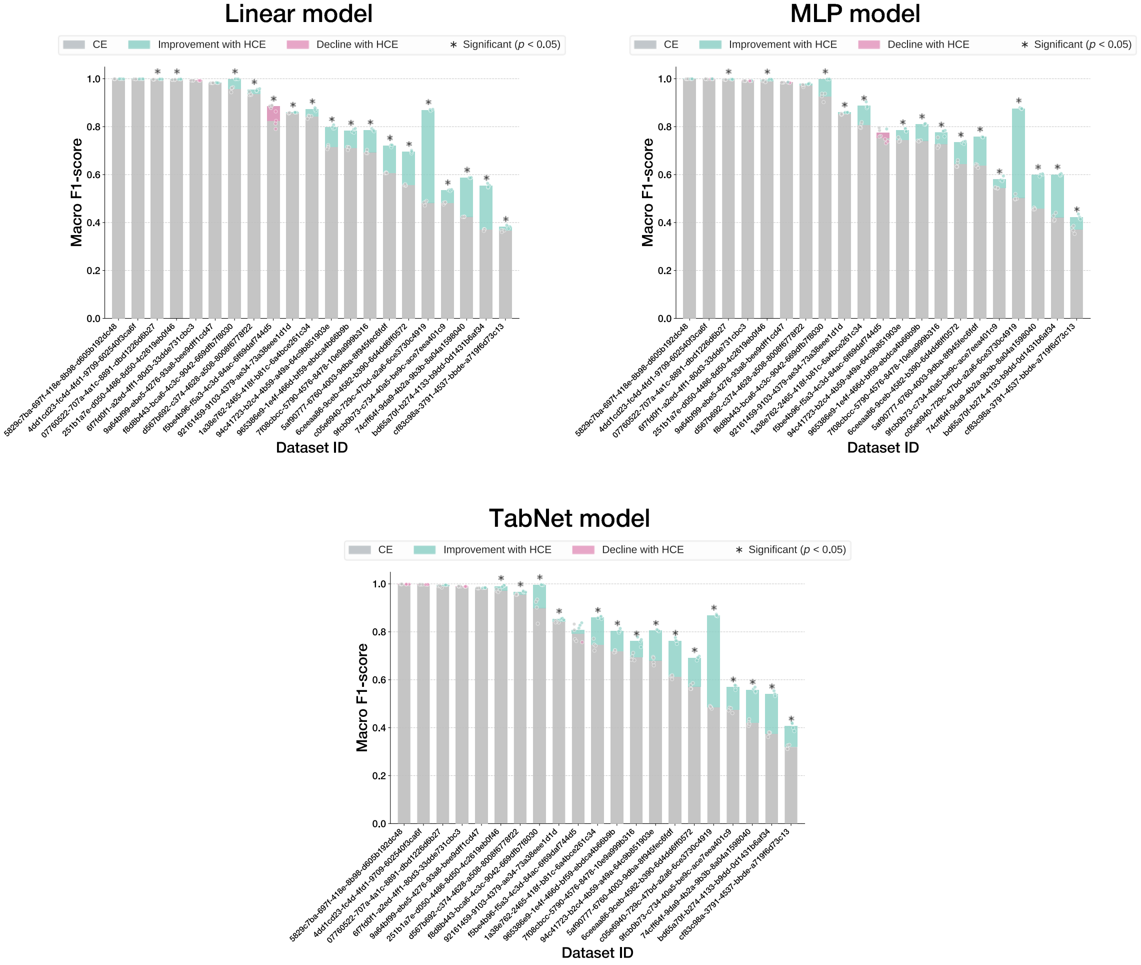

Supplementary Figure 1. Performance gains from the hierarchical cross-entropy (HCE) loss across 21 out-of-distribution test datasets for the linear classifier, multilayer perceptron (MLP), and TabNet. Improvements are measured relative to the same models trained with standard cross-entropy loss. All performance metrics reported reflect the mean over 4 independent training and evaluation runs per model, with results from each run shown as individual dots (color coding remains the same as in the legend). For each dataset, a paired t-test was performed and p-values were adjusted using the Holm–Bonferroni method to correct for multiple hypothesis testing.

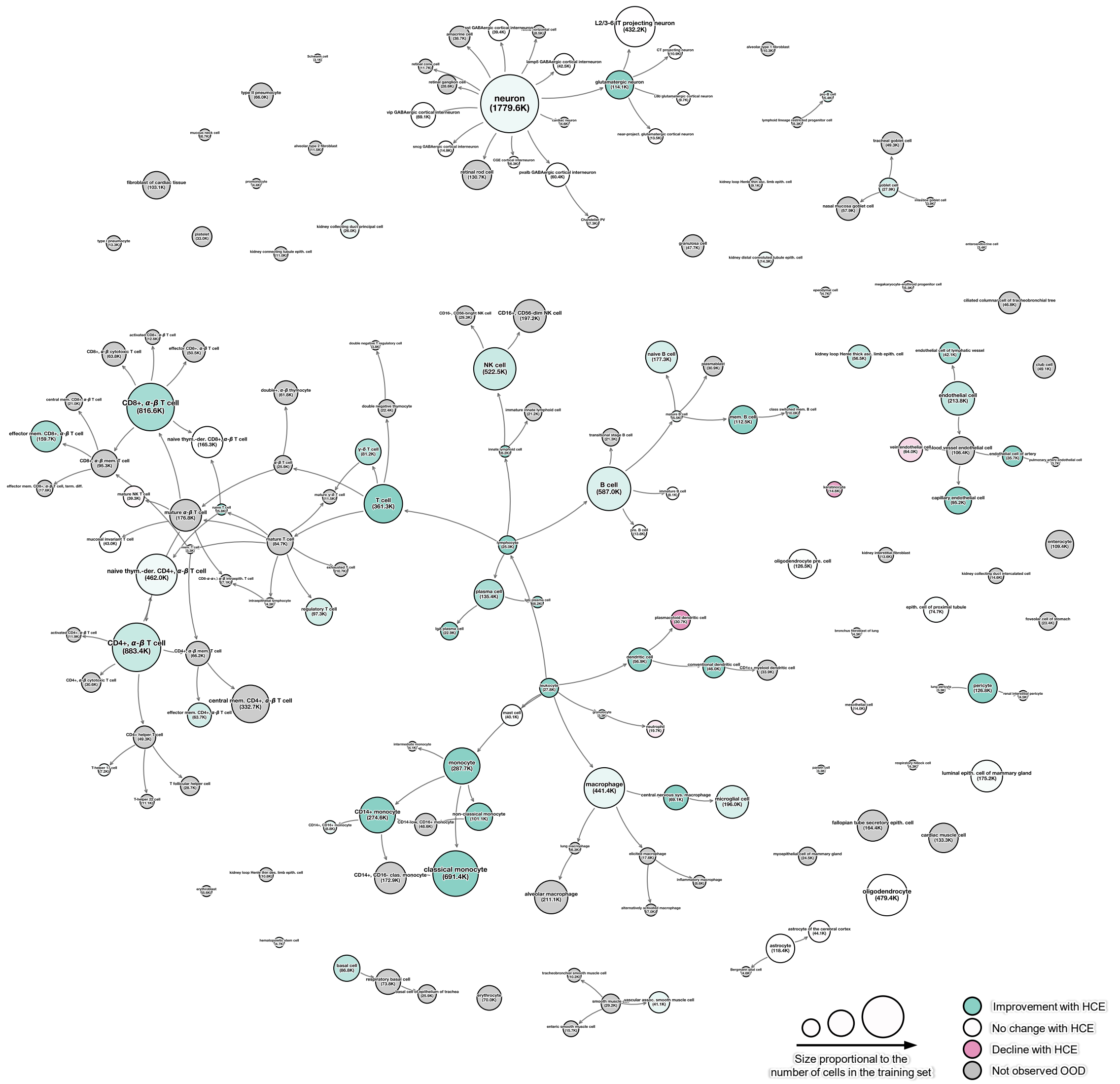

Supplementary Figure 2. Performance gains from the hierarchical cross-entropy (HCE) loss relative to standard cross-entropy, visualized on the cell ontology directed acyclic graph (DAG) for the linear model. Node size reflects the number of training examples per cell type; color indicates the change in F1-score (green for improvement, red for decline); grey nodes correspond to cell types not present in the out-of-distribution (OOD) test set. Note that this DAG consists of all 164 cell types seen in the training set.

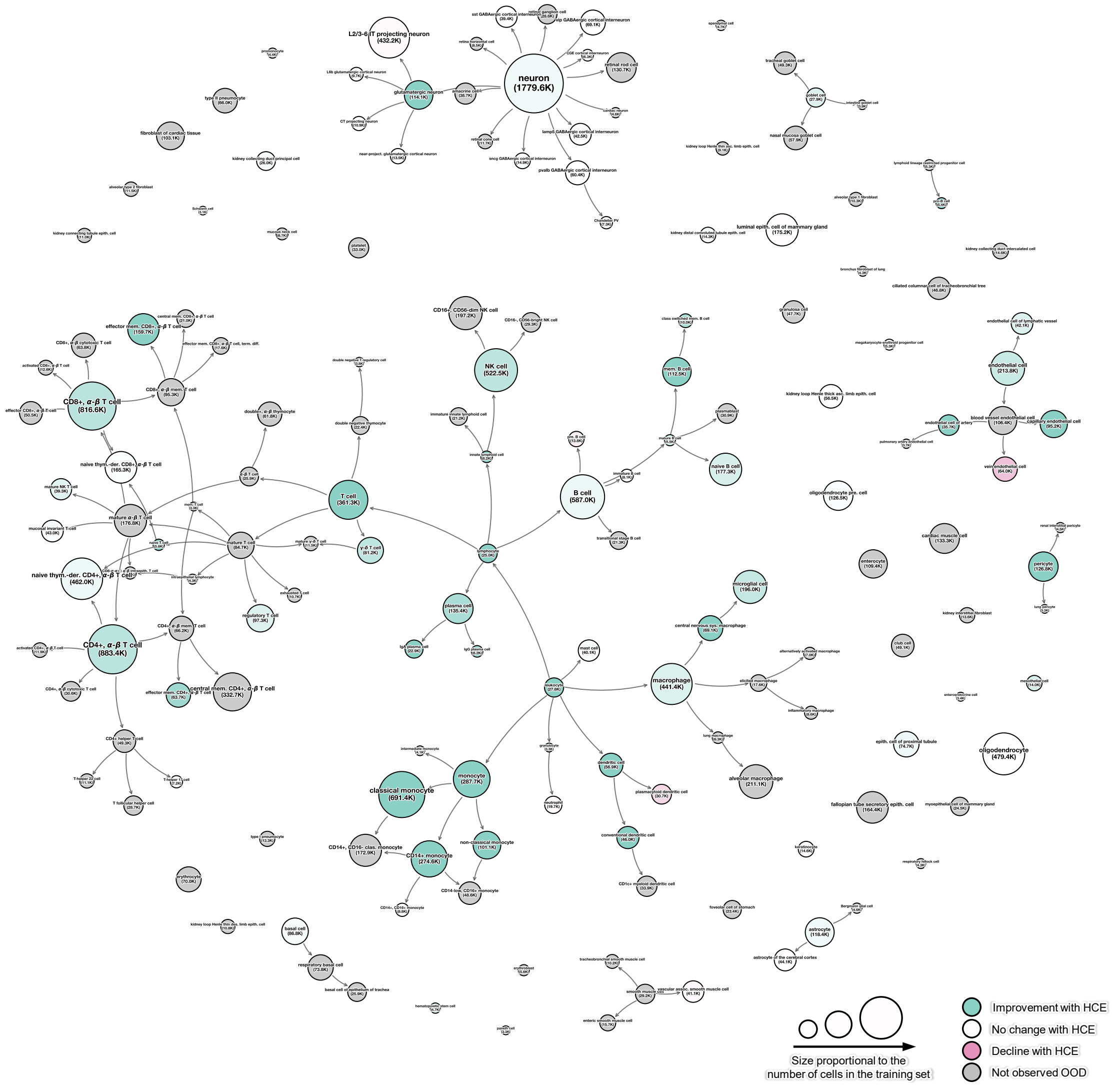

Supplementary Figure 3. Performance gains from the hierarchical cross-entropy (HCE) loss relative to standard cross-entropy, visualized on the cell ontology directed acyclic graph (DAG) for the MLP model. Node size reflects the number of training examples per cell type; color indicates the change in F1-score (green for improvement, red for decline); grey nodes correspond to cell types not present in the out-of-distribution (OOD) test set. Note that this DAG consists of all 164 cell types seen in the training set.

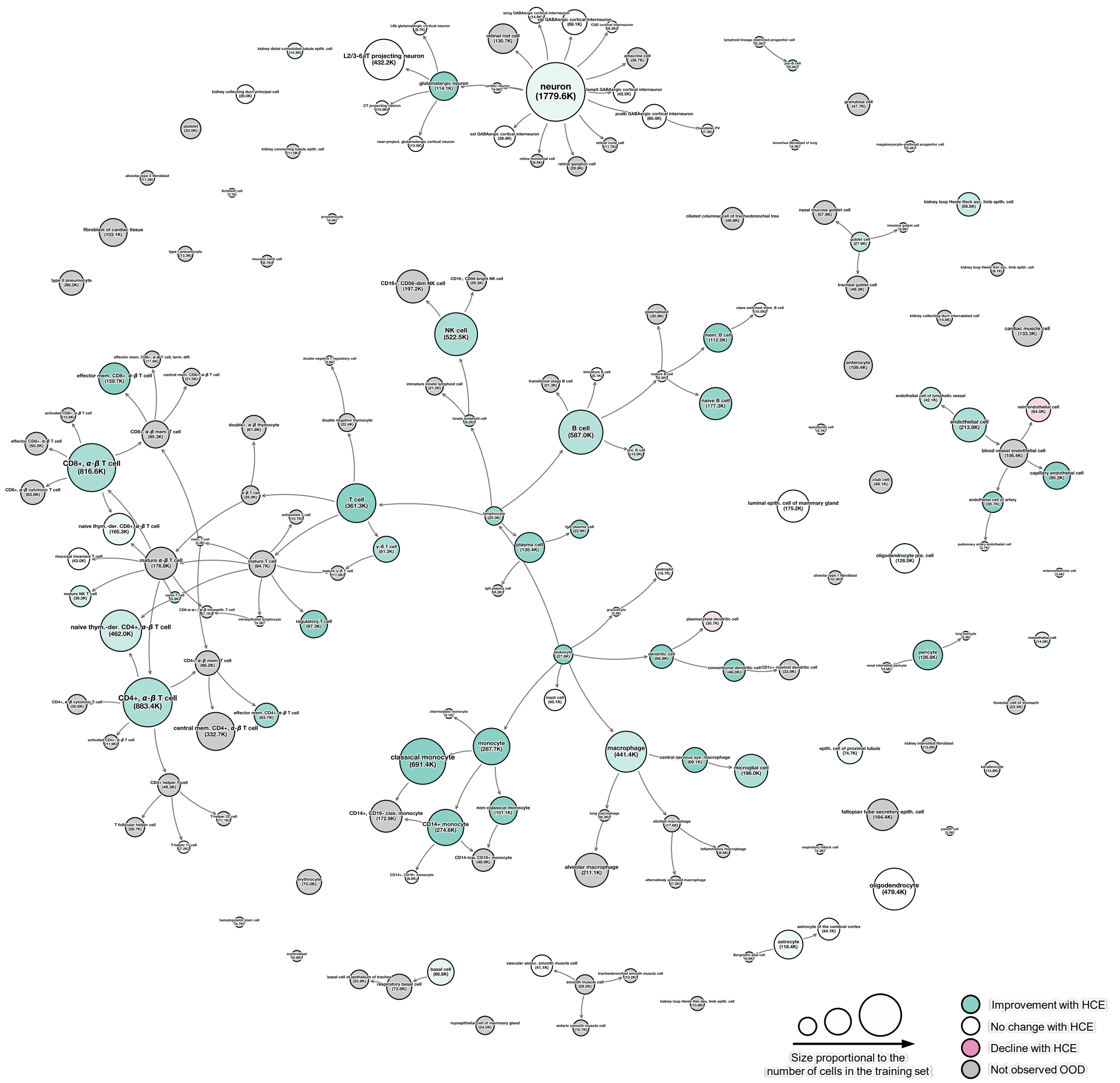

Supplementary Figure 4. Performance gains from the hierarchical cross-entropy (HCE) loss relative to standard cross-entropy, visualized on the cell ontology directed acyclic graph (DAG) for the TabNet model. Node size reflects the number of training examples per cell type; color indicates the change in F1-score (green for improvement, red for decline); grey nodes correspond to cell types not present in the out-of-distribution (OOD) test set. Note that this DAG consists of all 164 cell types seen in the training set.

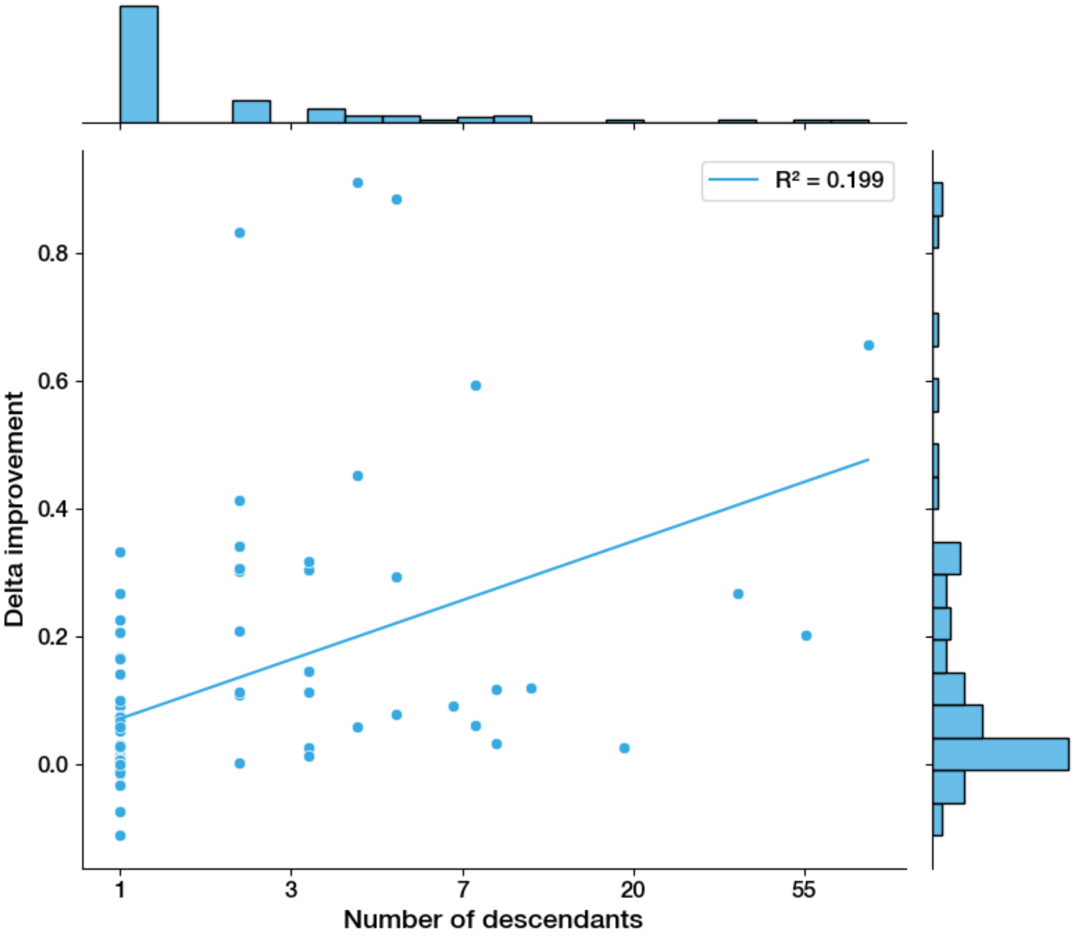

Supplementary Figure 5. Performance gains from the hierarchical cross-entropy loss in the multilayer perceptron (MLP) as a function of the number of descendants of a given cell type in the cell ontology directed acyclic graph (DAG). The number of descendants is reported in log scale base 10.

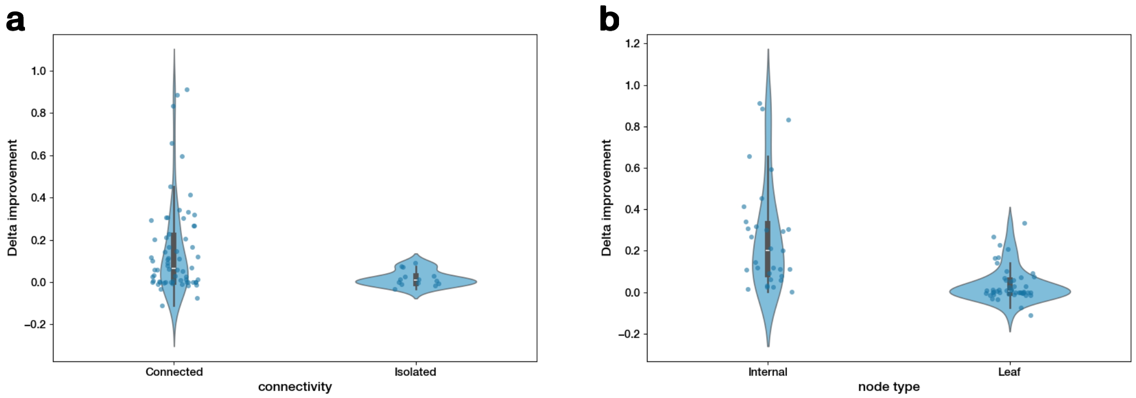

Supplementary Figure 6. Performance gains from the hierarchical cross-entropy loss in the multilayer perceptron (MLP) as a function of structural properties of the cell ontology directed acyclic graph (DAG). **a** Performance gains from the hierarchical loss for the MLP model on connected versus isolated nodes. **b** Performance gains from the hierarchical loss for the MLP model on internal nodes versus leaves.

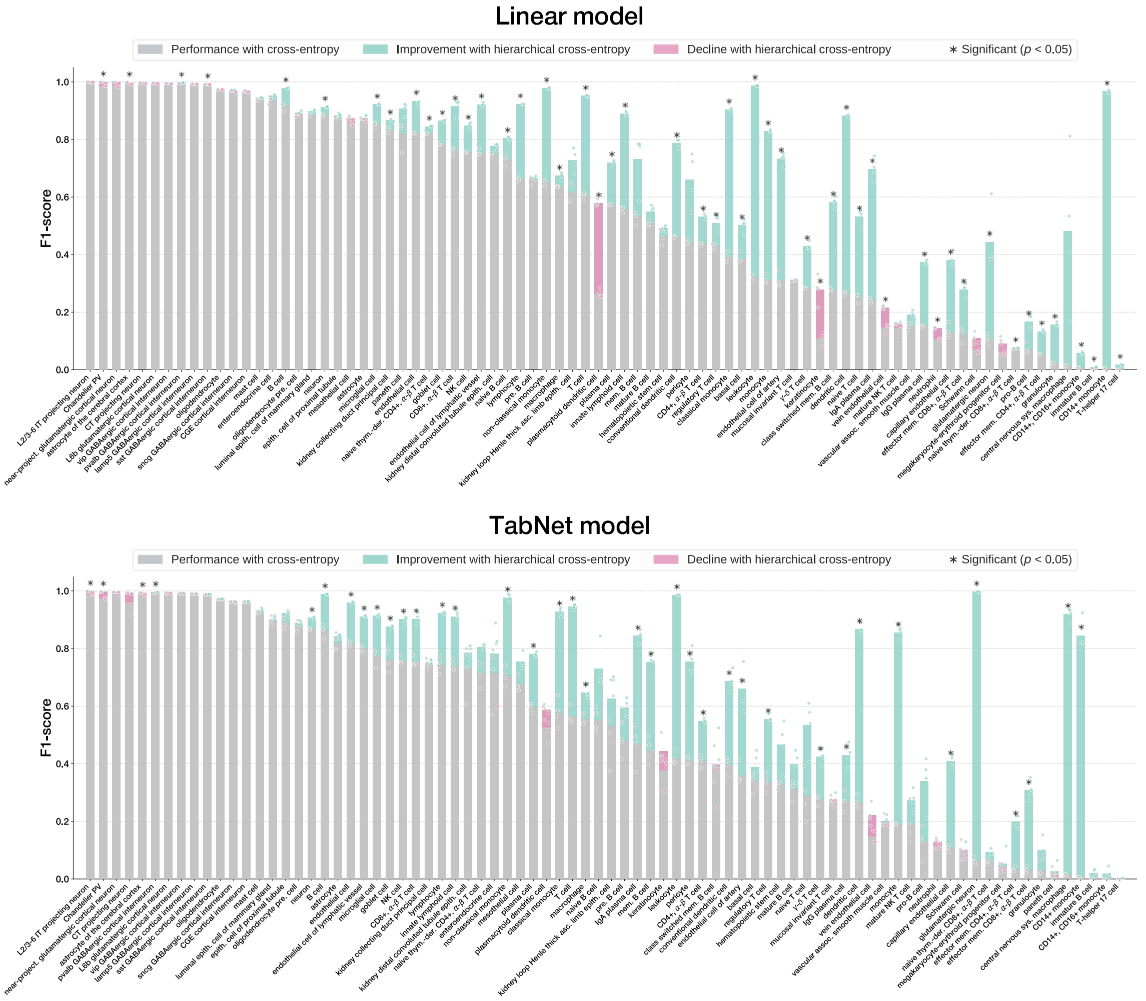

Supplementary Figure 7. Per–cell type performance changes induced by the hierarchical cross-entropy (HCE) loss strategy for the linear model and TabNet, shown relative to standard cross-entropy. All performance metrics reported reflect the mean over 4 independent training and evaluation runs per model, with results from each run shown as individual dots (color coding remains the same as in the legend). For each cell type, a paired t-test was performed and p-values were adjusted using the Holm–Bonferroni method to correct for multiple hypothesis testing.

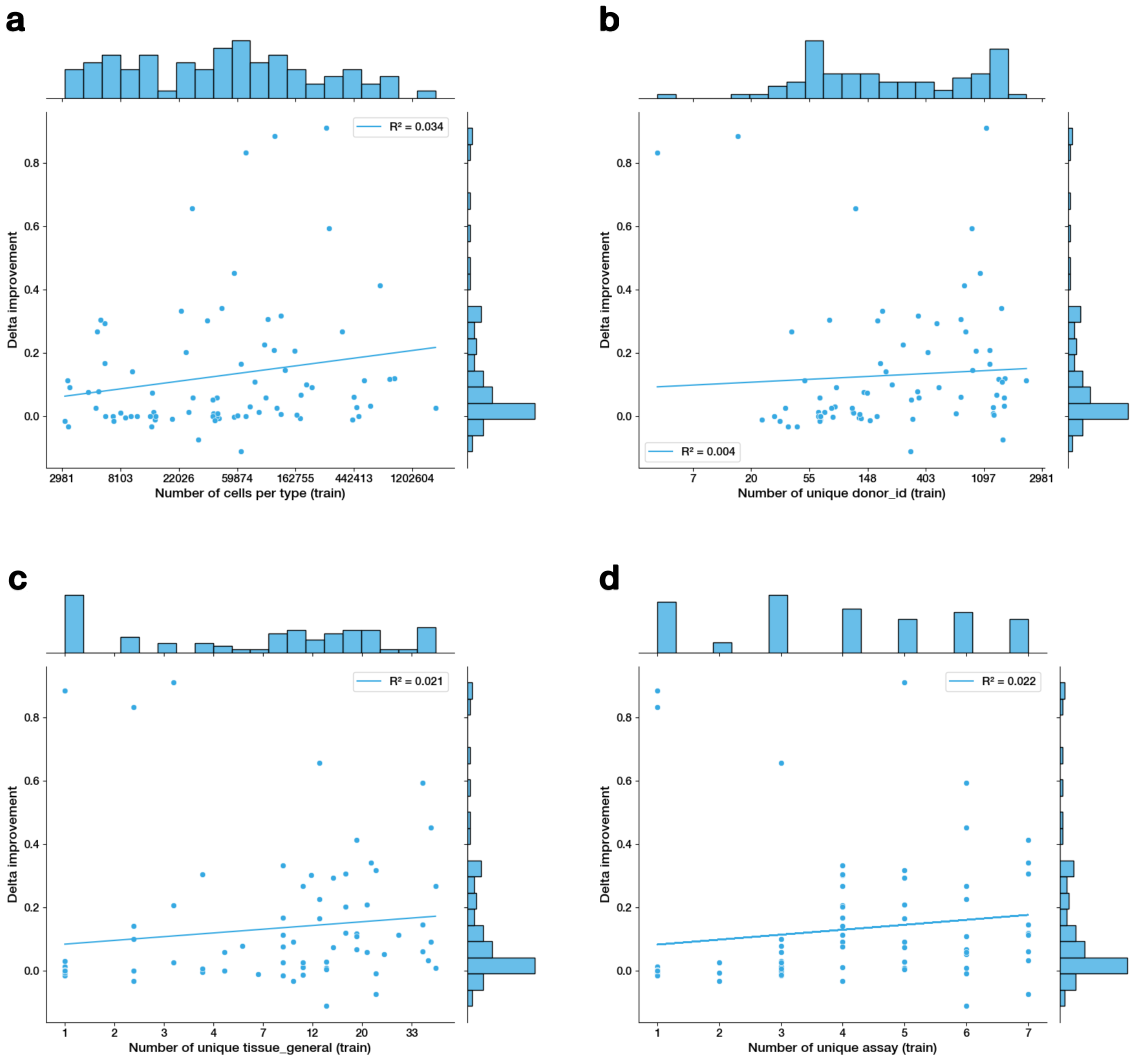

Supplementary Figure 8. Performance gains from hierarchical training in the multi-layer perceptron (MLP) model as a function of several training-set properties. These include: **a** cell type rarity (in log scale), **b** number of donors (in log scale), **c** number of tissues (in log scale), and **d** number of sequencing technologies (in linear scale).

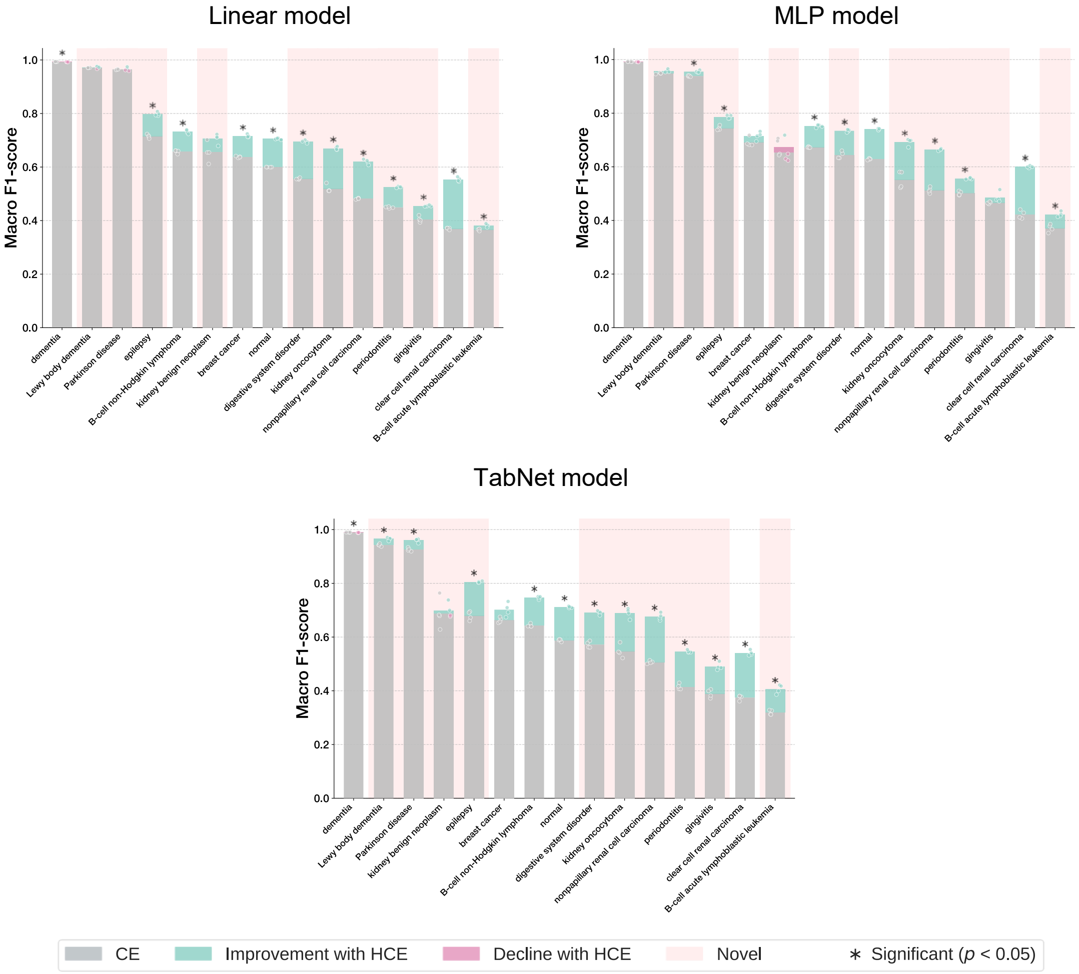

Supplementary Figure 9. Performance gains from the hierarchical cross-entropy (HCE) loss for different diseases for the linear classifier, multilayer perceptron (MLP), and TabNet. Improvements are measured relative to the same models trained with standard cross-entropy loss. Highlighted in pink are novel diseases in the test set that were not seen in the training set. All performance metrics reported reflect the mean over 4 independent training and evaluation runs per model, with results from each run shown as individual dots (color coding remains the same as in the legend). For each disease, a paired t-test was performed and p-values were adjusted using the Holm–Bonferroni method to correct for multiple hypothesis testing.

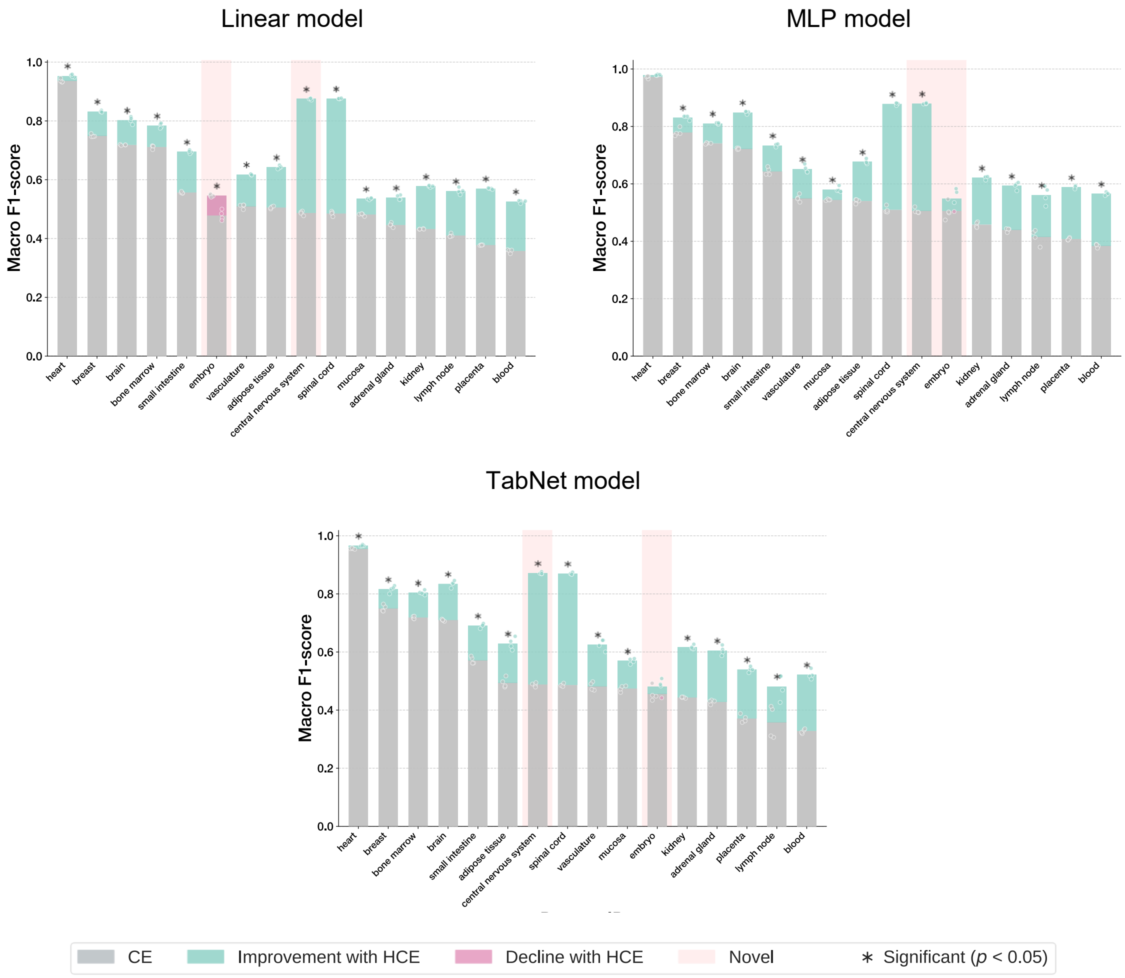

Supplementary Figure 10. Performance gains from the hierarchical cross-entropy (HCE) loss for different tissues for the linear classifier, multilayer perceptron (MLP), and TabNet. Improvements are measured relative to the same models trained with standard cross-entropy loss. Highlighted in pink are novel tissues in the test set that were not seen in the training set. All performance metrics reported reflect the mean over 4 independent training and evaluation runs per model, with results from each run shown as individual dots (color coding remains the same as in the legend). For each tissue, a paired t-test was performed and p-values were adjusted using the Holm–Bonferroni method to correct for multiple hypothesis testing.

Supplementary Tables

Supplementary Table 1. Values of the hyperparameters used to run the linear classifier. During training, the learning schedule was linear, the maximum learning rate was 0.0005, the optimizer was AdamW, and the weight decay parameter was set to 0.01.

Parameter	Value
**batch_size**	2048
**learning_rate**	0.0005
**learning rate scheduler**	torch.optim.lr_scheduler.StepLR<br>gamma = 0.9<br>step_size = 1 epoch
**optimizer**	AdamW
**weight_decay**	0.01
**augment_training_data**	False

Supplementary Table 2. Values of the hyperparameters used to run the multilayer perceptron (MLP). This model had 8 hidden layers (`n_hidden`) each with 128 neurons (`hidden_size`). During training, the learning schedule was linear, the maximum learning rate was 0.002, the optimizer was AdamW, the hidden layer dropout was 0.1, and the weight decay parameter was set to 0.05.

Parameter	Value
**batch_size**	2048
**learning_rate**	0.002
**learning rate scheduler**	torch.optim.lr_scheduler.StepLR<br>gamma = 0.9<br>step_size = 1 epoch
**optimizer**	AdamW
**weight_decay**	0.05
**n_hidden**	8
**hidden_size**	128
**dropout**	0.1
**augment_training_data**	True

Supplementary Table 3. Values of the hyperparameters used to run TabNet. This model has three main components: (1) a feature transformer, which is a multi-layer perceptron with batch normalization, (2) skip connections, and (3) a gated linear unit nonlinearity. The feature transformer maps the input gene expression data into a latent space of `n_a`+`n_d` dimensions, where the `n_a` = 64 portion is used to calculate attention masks and the `n_d` = 128 is used for cell type annotation. During training, the learning schedule was linear, the maximum learning rate was 0.005, the optimizer was AdamW, the feature attention mask is obtained by applying the 1.5-entmax function, and the weight decay parameter was set to 0.05.

Parameter	Value
**batch_size**	2048
**learning_rate**	0.005
**learning rate scheduler**	torch.optim.lr_scheduler.StepLR
	gamma = 0.9
	step_size = 1 epoch
**optimizer**	AdamW
**weight_decay**	0.05
**n_d**	128
**n_a**	64
**n_shared**	3
**n_independent**	5
**n_steps**	1
**lambda_sparse**	10^{-5}
**mask_type**	entmax
**virtual_batch_size**	256
**augment_training_data**	True
